# Hepatocellular Carcinoma Recurrence After Liver Transplantation: Current Insights and Future Directions

**DOI:** 10.3390/jcm14197009

**Published:** 2025-10-03

**Authors:** Ximena Parraga, Eyad Abdulrazzak, Ritah R. Chumdermpadetsuk, Marwan Alsaqa, Shanmukh Pavan Lingamsetty, Alan Bonder, Behnam Saberi

**Affiliations:** 1 Division of Gastroenterology and Hepatology, Beth Israel Deaconess Medical Center, Harvard Medical School, Boston, MA 02115, USA; ialabdul@bidmc.harvard.edu (E.A.); slingams@bidmc.harvard.edu (S.P.L.); abonder@bidmc.harvard.edu (A.B.); 2 Division of Transplantation, Department of Surgery, Beth Israel Deaconess Medical Center, Harvard Medical School, Boston, MA 02115, USA; rchumder@bidmc.harvard.edu; 3 Division of Internal Medicine, Saint Peter’s University Hospital, Rutgers Robert Wood Johnson Medical School, New Brunswick, NJ 08901, USA; malsaqa@saintpetersuh.com

**Keywords:** hepatocellular carcinoma, liver transplantation, recurrence, immunotherapy

## Abstract

Hepatocellular carcinoma (HCC) is a leading cause of cancer death, with liver transplantation (LT) offering a curative option for early-stage patients who cannot undergo resection. Although LT provides good long-term outcomes within standard criteria, recurrence occurs in approximately 8–20% of recipients and often leads to poor survival. Traditionally, LT eligibility relied on strict criteria like the Milan criteria, which are effective in selecting patients with low recurrence but may exclude patients who could benefit from transplantation. In response, new expanded criteria and models using tumor biology have been developed for better risk stratification, allowing more personalized selection and management. Despite these advances, recurrence remains a major clinical challenge, with no consensus on optimal imaging timing or frequency post-LT. Treatment depends on the recurrence’s extent and location, including surgical resection and locoregional therapies. Systemic treatments are promising, especially for unresectable or extrahepatic recurrence, though most evidence comes from small retrospective studies, limiting the development of standardized protocols. Future research should focus on addressing these gaps and guiding evidence-based post-transplant care. This is a narrative review summarizing recent advances in HCC recurrence.

## 1. Introduction

Hepatocellular carcinoma (HCC) represents one of the highest burdens of cancer-related mortality worldwide [1], ranking as the third leading cause of cancer-related death [1]. For patients with early-stage HCC who are not candidates for resection due to liver dysfunction or multifocal tumors, liver transplant (LT) offers a curative option for both the cancer and the underlying liver disease [2]. While LT remains the preferred treatment for early-stage HCC within the standard criteria [3], recurrence remains a major concern, occurring in approximately 7–20% of recipients and associated with poor outcomes, with a median survival of around one year [3,4,5,6,7,8,9].

In the United States, the incidence of HCC has increased in recent decades, historically driven by hepatitis C virus (HCV) infection [10]. However, with the widespread adoption of direct-acting antivirals, the incidence of HCV-related HCC has declined. In contrast, metabolic dysfunction–associated steatotic liver disease (MASLD) has emerged as a rising etiology due to the growing obesity in the US [11]. This trend suggests that MASLD may soon become the leading cause of HCC requiring transplantation [11]. This is particularly concerning, as MASLD has been associated with worse graft survival outcomes [12]. At the same time, the increasing demand for LT continues to outpace organ availability, prompting the broader use of expanded criteria donors and living donor liver transplants (LDLT) [13]. These shifts, alongside the risk of HCC recurrence, underscore the importance of optimizing candidate selection to balance the benefits of transplantation with the risk of post-LT recurrence [4].

The Milan criteria (MC) have long been the benchmark for LT eligibility [4,14]. While LT within MC is associated with excellent post-LT survival [15], some patients who can benefit from LT may be excluded [16], as they do not meet the criteria. It is important to note that overall criteria that solely rely on tumor size and number, such as the MC, have their limitations in predicting HCC recurrence post-LT [17]. Recognition of the limitations of size-based criteria prompted the development of prognostic models that integrate tumor biology and biomarkers [18] to better risk-stratify HCC recurrence after LT [4,19]. One example is the Canadian Extended Toronto Criteria, which incorporates pre-transplant biopsy to assess tumor differentiation, placing greater emphasis on tumor biology [20]. By capturing dynamic and biologically relevant features, these models offer a more individualized approach to transplant candidacy, post-LT surveillance, and potential adjuvant strategies [18,21].

There is a pressing need for selection criteria that integrate both tumor volume and biomarkers [18] reflective of tumor biology [21] to more accurately assess the recurrence risk of tumor recurrence following LT while expanding access to transplantation. This need has driven the development of risk-stratification models designed to predict post-transplant recurrence. Nevertheless, further research is needed to evaluate their long-term effectiveness, as optimal patient selection remains essential for achieving favorable outcomes [22]. In addition, understanding other donor and recipient risk factors associated with post-LT HCC recurrence is of importance. This narrative review will focus on the recent literature regarding HCC recurrence. We aim to discuss risk factors, candidate selection, surveillance strategies, and treatment approaches for HCC recurrence after LT, with a focus on improving post-transplant outcomes through individualized care.

## 2. Understanding Recurrence: Who Is at Risk?

To understand the complexity of recurrence after LT and identify patients at the highest risk, it is essential to examine the complex interplay of predictive factors. While traditional morphological features, such as tumor size and number, are well-established risk factors, there is increasing interest in including factors such as tumor differentiation, vascular invasion, and other markers that could reflect biology.

### 2.1. Tumor Biology: Beyond Morphology

While tumor volume (size and number of nodules) has long served as the foundation for LT selection criteria, growing evidence highlights the need to shift from size-based only models toward incorporating biology-based factors. Although both tumor size and number are independently associated with recurrence, larger tumors are more consistently linked to adverse outcomes, likely due to their greater propensity for vascular invasion and intratumoral heterogeneity [23,24,25]. For instance, tumors ≥ 10 cm are associated with a fourfold increase in post-transplant recurrence or mortality [23]. In clinical practice, these morphological criteria remain central to patient eligibility. In the U.S., the MC and University of California San Francisco (UCSF) criteria, which are based on tumor number and size, are the most widely applied. The MC defines eligibility as a single HCC no larger than 5 cm, or up to three nodules, each measuring no more than 3 cm [26]. The UCSF criteria include patients with a single tumor measuring ≤6.5 cm, or up to three nodules with the largest ≤4.5 cm and a total tumor diameter ≤8 cm [27]. However, as mentioned earlier, their predictive accuracy is limited, with reported AUCs of 0.63 and 0.65, respectively [28]. In contrast, Canada’s Extended Toronto Criteria incorporate biopsy-based differentiation and symptomatology, reflecting a more nuanced approach to tumor biology [20]. While encouraging results have been reported using a large cohort of patients, it remains a single-center experience. Prospective multicenter validation remains lacking, which limits their reproducibility and generalizability across diverse patient populations [17]. It is known that other factors that are reflective of tumor biology are associated with worse survival [29] and are essential in the prediction of HCC recurrence following liver transplant, such as poor tumor differentiation and microvascular invasion [29]. However, this information is not available at the time of transplant in most cases, as pre-LT biopsy of the HCC is rarely done. In addition, even with the pre-transplant biopsy of the lesions, the results may vary based on tumor heterogeneity [30].

Other expanded frameworks include the Up-to-7 (U7), Kyoto Criteria (KC), Asan Criteria (AC), and Hazard Associated with Liver Transplantation for HCC (HALT) Criteria. These have been proposed to extend transplant eligibility by integrating tumor burden with biological markers and microvascular invasion. The U7 criteria, introduced in 2009, define eligibility when the sum of the largest tumor diameter (cm) and number of nodules is ≤7. In patients without microvascular invasion, 5-year survival (71.2%) was comparable to Milan (73.3%), supporting its role as a safe expansion in selected candidates [31]. In 2024 national analysis found that, compared with the Milan criteria, using U7 allowed a 3.5% expansion in transplant candidacy with only a 1.5% decrease in 3-year overall survival, while HALT-HCC and Metroticket 2.0 offered superior recurrence prediction [32]. The Kyoto criteria, proposed in 2009, define eligibility for LT in HCC patients as ≤10 tumors each ≤5 cm in diameter, with a PIVKA-II level ≤400 mAU/mL. These criteria reported remarkably favorable outcomes, 5-year recurrence of only 3% and survival rate of 87% among qualifying patients [33]. The AC expands transplant eligibility to patients with up to six nodules (largest ≤ 5 cm) and no gross vascular invasion. In 2015, a validation study demonstrated that AC identified more LT candidates than Milan or UCSF, while patients meeting AC presented comparable 5-year overall and disease-free survival to those meeting Milan or UCSF [34]. More recently, in 2017, the HALT-HCC criteria were introduced, which consider tumor burden score, AFP levels, and MELD-Na. It was validated in large U.S. cohorts, it stratifies prognosis both within and beyond MC, showing better predictive performance than traditional criteria [35].

### 2.2. Biomarkers in Risk Stratification

Serum biomarkers are emerging as powerful tools to capture tumor aggressiveness more precisely than morphology alone. AFP remains the most established biomarker, and high pre-transplant AFP levels may reflect tumor biology and are consistently associated with higher recurrence risk [36,37]. In 2016, the United Network for Organ Sharing (UNOS) introduced a policy excluding candidates with AFP > 1000 ng/mL [38], and for patients undergoing downstaging with locoregional therapy (LRT), eligibility requires AFP < 500 ng/mL [38]. However, AFP remains a suboptimal biomarker for predicting tumor recurrence. Its levels can be confounded by liver regeneration, inflammation, chronic hepatitis, and even other malignancies, while remaining within the normal range in over half of HCC patients, depending on the threshold applied. These limitations significantly reduce its reliability as a standalone marker for post-LT risk stratification [28]. This limitation highlights the pressing need for the development of novel tumor biomarkers, as we need novel tumor biomarkers in the field of HCC. PIVKA-II (des-γ-carboxy prothrombin) and inflammatory markers such as a neutrophil-to-lymphocyte ratio (NLR) ≥5 have also demonstrated prognostic value [36,39,40,41]. Other hematological markers, such as the platelet-to-lymphocyte ratio (PLR) have increasingly been recognized with recurrence risk. Prior meta-analysis emphasized that higher preoperative PLR predicts poorer recurrence-free survival (RFS) and disease-free survival (DFS) [42,43]. In Addition, Extracellular matrix (ECM) turnover markers, such as type III procollagen peptide, and genetic biomarkers, including specific gene expression signatures, have demonstrated utility in refining risk prediction for HCC recurrence, Notably, HCCs with high intratumor fibrosis and expression of WNT/TGFB subclass signatures exhibit worse prognosis, supporting the clinical relevance of ECM markers in recurrence prediction [44]. MicroRNAs, particularly circulating and exosomal forms, are emerging as promising biomarkers of recurrence following LT. High levels of exosomal miR-21 and miR-221 have been linked to higher recurrence risk and poorer prognosis, while changes in circulating miR-122 are often utilized for diagnosis and post-transplant surveillance. These changes often appear earlier than AFP or DCP, making them useful for earlier surveillance [45,46,47]. These biomarkers stratify patients with greater recurrence risk and poor survival following LT, underscoring their potential for biology-based selection, but their clinical use awaits multicenter validation.

### 2.3. Vascular Invasion

Vascular invasion is one of the strongest predictors of recurrence and is typically classified as macrovascular (visible on imaging) or microvascular (only confirmed post-explant). Macrovascular invasion remains a contraindication for LT in most guidelines [14], though some patients may become eligible after successful downstaging and showing stability following locoregional therapy [48]. Microvascular invasion (MVI) is also associated with worse post-transplant outcomes, even among patients who meet standard criteria like Milan [49,50]. Predicting MVI preoperatively remains a challenge. Features such as large tumor size, irregular margins, and elevated PIVKA-II levels may suggest its presence [51]. Recent models integrating radiologic and radiomic features have shown promise, though standardized prediction tools are still under development [52].

### 2.4. The Role of Graft Type

Graft-related factors, particularly the type of donor, have also been investigated. Living donor liver transplants (LDLT) provide a valuable alternative for patients who might be ineligible for transplantation due to the shortage of deceased donor organs [13,53]. Early studies raised concerns about higher recurrence rates in LDLT recipients compared to those receiving deceased donor liver transplants (DDLT), potentially due to the regenerative stimulus in partial grafts promoting tumor growth [54]. However, subsequent research has shown that overall survival in LDLT recipients is comparable and, in some cases, superior to DDLT [55,56]. Furthermore, recurrence appears to be more closely linked to tumor biology than to graft type per se [57]. A meta-analysis reported no significant difference in recurrence rates between LDLT and DDLT [58]. One explanation for earlier findings may be the broader use of expanded selection criteria in LDLT. To mitigate this, a waiting period of 6 months has been proposed before proceeding with transplant to allow a better assessment of tumor behavior and biological aggressiveness [59].

A higher donor risk index (DRI) driven by factors such as advanced donor age, diabetes, obesity, and severe graft steatosis has been consistently associated with increased recurrence of HCC after liver transplantation, likely due to greater graft injury and the need for intensified immunosuppression [60]. The use of split or partial liver grafts does not independently increase HCC recurrence in recipients who meet standard selection criteria, but in higher-risk populations (e.g., those outside Milan criteria or with small-for-size grafts), recurrence rates may be elevated, reflecting the interplay between graft quality and tumor biology [61,62]. Immunological compatibility, particularly ABO incompatibility and higher degrees of HLA mismatch, can contribute to increased immunosuppression requirements and has been linked to higher recurrence risk, although the impact is less pronounced than donor and tumor factors [63].

## 3. Shaping Eligibility: The Role of Downstaging and Selection Criteria

Downstaging before transplantation has become a crucial strategy to expand access to LT for patients beyond the standard criteria [64]. By reducing tumor burden using LRT, in initially ineligible patients, such as those with MVI, a known contraindication due to high recurrence risk, studies show improved long-term outcomes. Recently, Mehta et al. proposed expanding conventional downstaging criteria to include patients who are outside the UNOS downstaging parameters, provided they can be successfully downstaged within the Milan criteria. And that the use of systemic therapy following successful downstaging could further improve eligibility for transplant [64]. A retrospective multicenter study assessed outcomes in patients who underwent downstaging until achieving complete radiological regression, reporting an acceptable recurrence rate (11%) [48]. A retrospective analysis of the UNOS database compared post-transplant outcomes among patients initially beyond Milan criteria, stratified into two groups: (1) those who met the UNOS downstaging (UNOS-DS) criteria and (2) those who did not (all-comers, AC-DS), alongside a reference group of patients within Milan criteria. The study found comparable 3-year post-LT survival between the Milan and UNOS-DS groups; however, the AC-DS group demonstrated significantly lower survival outcomes [65]. Additionally, a meta-analysis published by Tan et al. demonstrated the clinical validation for the use of UNOS-DS criteria [66].

To further assess tumor biology and ensure adequate, durable responses, a 6-month waiting period after successful downstaging treatment is established before granting a Model for End-Stage Liver Disease (MELD) exception [67]. During this period, patients are monitored for disease progression or recurrence, allowing for an evaluation of tumor behavior. Under the Median MELD at Transplant minus 3 (MMaT-3) rule, patients who complete this waiting period are assigned a MELD exception score three points lower than the regional median MELD at transplant. This approach helps prioritize candidates with favorable tumor biology [68].

## 4. Managing the Balance: Immunosuppression and Oncologic Risk

Immunosuppressive therapy post-LT may influence the risk of HCC recurrence through modulation of immune surveillance and tumor-promoting pathways [69]. Calcineurin inhibitors (CNIs), such as tacrolimus and cyclosporine, are part of the standard immunosuppressive regimens. However, it has been associated with increased tumor progression. High early CNI exposure, especially during the first month, has consistently been linked to a higher recurrence risk, likely due to both impaired immune control and activation of tumorigenic pathways such as TGF-β [70,71]. Observational evidence links higher CNI exposure to an increased risk of HCC recurrence [72]. Similarly, in a separate cohort, mean tacrolimus trough levels ≥10 ng/mL during the first month post-transplant were associated with a fivefold higher 5-year recurrence rate (50% vs. 9.1%) [73]. A later analysis of 219 HCC patients transplanted within Milan criteria demonstrated that elevated CNI exposure during the first month post-LT, mean tacrolimus trough levels >10 ng/mL or cyclosporine >300 ng/mL, was independently associated with an increased risk of HCC recurrence (27.7% vs. 14.7% at 5 years; *p* = 0.007), whereas CNI exposure beyond this period was not [74]. This has prompted interest in CNI-sparing strategies, especially during the early post-transplant period.

Mammalian target of rapamycin (mTOR) inhibitors (e.g., sirolimus, everolimus) are often used as immunosuppressive agents post-LT. Opposite to CNIs, mTOR inhibitors are linked to anti-tumor properties. Mechanistically, they suppress tumor cell proliferation, angiogenesis, and protein synthesis, processes often upregulated in HCC [75]. A large registry-based analysis demonstrated that sirolimus-based therapy was independently associated with improved survival after LT for HCC but not for non-HCC indications, suggesting a cancer-specific benefit [76]. A more rigorous follow-up study using linked registry and pharmacy data found no mortality benefit to sirolimus exposure compared to no exposure [77]. Numeric reductions in HCC recurrence and cancer-specific mortality were observed but not statistically significant (aHR 0.86, 95% CI 0.45–1.65; aHR 0.80, 95% CI 0.43–1.50, respectively) [77]. The SiLVER trial, for instance, found no long-term improvement in recurrence-free survival with sirolimus, though early benefits in high-risk patients (e.g., those with elevated AFP) were observed. Later analyses indicated that sirolimus use for ≥3 months may confer survival advantages in biologically aggressive tumors. However, these possible benefits must be weighed against increased risks of acute rejection and poor wound healing [78]. A meta-analysis by Zhang et al. highlighted the importance of mTOR inhibitors in reducing the risk of HCC recurrence compared to CNI [79].

The role of mycophenolate mofetil (MMF) has been examined in post-transplant HCC recurrence. In a retrospective cohort study of 1250 liver transplant recipients with HCC in Taiwan, MMF administration was associated with a significantly higher recurrence rate, particularly at higher cumulative doses, whereas low-dose MMF did not increase recurrence risk [80]. However, there is reported potential of antitumor effects of Mycophenolic Acid (MPA) in vitro and reduced HCC recurrence in liver transplant patients [81]. These contrasting findings indicate the need for further investigation.

The impact of other components of standard immunosuppressive regimens on HCC recurrence remains less well-defined. Corticosteroids, which exert broad immunosuppressive effects, are central to preventing acute rejection. However, concerns persist regarding their potential to promote tumor progression by dampening immune surveillance, suppressing neutrophil-mediated tumor cytostasis, and facilitating tumor migration [82]. Data on their oncologic impact are mixed: some studies suggest improved recurrence-free and overall survival with early steroid withdrawal, while others show no significant difference [83]. Similarly, evidence regarding commonly used induction agents, anti-thymocyte globulin (ATG) and basiliximab is limited and inconclusive concerning their impact on HCC recurrence [4]. Overall, optimizing immunosuppression in LT recipients with HCC remains a delicate balance between oncologic control and graft preservation.

## 5. Recurrence Patterns, Prognosis, and Surveillance

A meta-analysis of 1021 patients who presented recurrence after undergoing LT found that recurrence most commonly occurs at extrahepatic locations, accounting for approximately two-thirds of the patients, with or without intrahepatic involvement [84]. The most common sites are the lungs, followed by bone, adrenal glands, lymph nodes, and, rarely, the brain. Intrahepatic-only recurrence accounts for 10–30% of cases [84,85,86].

### 5.1. Early Recurrence

Early recurrence is defined as within the first 2 years, accounting for ~70% of all recurrences, and is associated with a poorer prognosis when compared to late recurrence [87,88,89]. It is typically driven by aggressive tumor biology, such as larger tumor burden, MVI, and poorly differentiated lesions. One theory suggests early recurrence results from occult metastases present at the time of LT, potentially disseminated through portal circulation or circulating tumor cells (CTCs), which may remain dormant and later activate [90]. However, the prognostic utility of CTCs remains limited, highlighting the need for further investigation.

### 5.2. Late Recurrence

Late recurrence is defined as recurrence occurring after 2 years, and it is often linked to de novo HCC [91] and associated with chronic liver disease, cirrhosis, older recipient age, and hepatitis B infection [88]. It tends to be intrahepatic and generally carries a better prognosis.

### 5.3. Surveillance

Given that early recurrence is associated with worse outcomes, surveillance is critical during the first 1–2 years after LT [92]. Studies suggest more frequent imaging, such as every 3–6 months, can improve post-recurrence survival [92]. The RETREAT score, which incorporates explant pathology and tumor biology, is a validated tool to guide surveillance intensity [93,94]. Recommended protocols depend on the score: score 1–3: CT chest/abdomen + AFP every 6 months (for the first 2 years), Score 4: same every 6 months, extended beyond 2 years, Score ≥ 5: every 3–4 months (for the first 2 years), then every 6 months (up to year 5) [94].

Despite the high rate of extrahepatic recurrence, routine imaging of bones and pelvis is not commonly performed. The American Association for the Study of Liver Diseases (AASLD) recommends chest and abdominal CT or MRI, but no consensus exists on the ideal frequency or imaging scope [2]. Broader imaging (e.g., pan-scan CT or PET scan) may improve detection but needs further validation.

AFP is commonly used in surveillance protocols but is limited in sensitivity, as only a subset of tumors secrete AFP [95]. Underscoring once again the need for better biomarkers. Beyond immunosuppressive strategies, there is growing interest in leveraging tumor genomics and novel biomarkers to enable earlier detection of recurrence and to personalize post-transplant care. Techniques and biomarkers such as CTCs, circulating tumor DNA (ctDNA), and extracellular RNA offer minimally invasive methods to identify molecular signatures associated with recurrence risk [96,97,98]. Emerging studies suggest that detecting ctDNA or CTCs in the bloodstream can precede radiographic evidence of relapse, providing a potential window for earlier intervention [99]. An experimental study identified and characterized small RNA clusters (smRCs) within extracellular vesicles (EVs), demonstrating their potential as minimally invasive biomarkers for early surveillance of HCC [98]. Additional investigational approaches include transcriptomic profiling to identify high-risk gene expression signatures, combined biomarker panels (ctDNA, exosomal RNA, proteins), and single-cell sequencing to characterize tumor heterogeneity and immune landscape [98,100,101]. An experimental study using gene expression profiling in formalin-fixed, paraffin-embedded (FFPE) liver tissue found that a gene signature in adjacent non-tumoral tissue could predict survival outcomes and late recurrence, which is often associated with the development of de novo tumors.

While promising, these technologies require further clinical validation. The 2020 International Liver Transplantation Society (ILTS) consensus acknowledged the absence of standardized surveillance strategies and recommended individualized follow-up based on recurrence risk [91,102,103].

## 6. Treatment Strategies: From Curative to Palliative

Various treatment strategies have been explored for managing post-LT HCC recurrence, including surgical resection, locoregional therapies, and, more recently, systemic treatments. Several factors must be considered when selecting the most appropriate approach. The choice of treatment should be guided by the location of the recurrence, the use of previous treatments, the timing and extent of recurrence following LT [84], as well as the distinction between curative and palliative intent [104]. While systemic therapies represent a promising approach, most of the current evidence comes from small retrospective studies and case series [84]. This limits the generalizability of findings and contributes to uncertainty in defining optimal treatment pathways. In Table 1, we summarize the current treatment strategies based on the location of recurrence. And in Table 2, we summarize the recurrence outcomes by treatment.

### 6.1. Intrahepatic

In cases of intrahepatic-only recurrence, surgical resection remains the preferred curative-intent treatment for eligible patients and has demonstrated favorable survival outcomes [5]. This approach is most appropriate for patients with localized disease or solitary lesions, absence of vascular invasion or distant metastases, and preserved liver graft function [105]. However, a significant proportion of patients present with more advanced disease at the time of recurrence, which often limits the feasibility of resection. A recent systematic review and meta-analysis found that, among patients with single-nodule HCC recurrence, surgical resection was superior to LRTs in one-year overall survival (71% vs. 62%, *p* = 0.038) [104].

Thermal ablation (TA) is another curative-intent treatment option for HCC, particularly for intrahepatic recurrences. While much of the evidence comes from studies not specific to post-transplant recurrence, TA has demonstrated efficacy in this setting as well. It has shown comparable survival outcomes to surgical resection in patients who are not candidates for surgery. A retrospective study further supported its role by reporting favorable outcomes in both intrahepatic and pulmonary HCC recurrences after LT [106].

For unresectable intrahepatic HCC, transarterial chemoembolization (TACE) remains one of the primary therapeutic options [107]. In the setting of post-transplant recurrence, a retrospective study demonstrated that TACE was associated with a survival benefit in patients with multiple intrahepatic recurrences [124]. Combination approaches have also shown promise; for example, TACE combined with thermal ablation has been associated with improved survival compared to TACE alone [116].

Yttrium-90 (Y90) radioembolization is mostly used in the pre-transplant setting for downstaging, where it has demonstrated excellent outcomes with low recurrence rates [126], or as a palliative treatment in select patients with recurrent intrahepatic HCC who are ineligible for surgery or ablation [127]. However, evidence supporting its use for treating HCC recurrence remains scarce [108]. A retrospective study with 41 patients with HCC recurrence following surgical resection reported that treatment with Y-90 radioembolization was associated with a median time to progression (TTP) of 11.3 months and a median overall survival (OS) of 22.1 months [128]. While these findings are encouraging, most available data come from post-resection settings. Further studies are needed to clarify the potential role and benefit of Y-90 radioembolization in patients with recurrent HCC, particularly in the post-transplant setting.

### 6.2. Extrahepatic

Extrahepatic recurrence accounts for a significant proportion of post-transplant HCC recurrence, with the lungs being the most frequent location of metastasis [115]. Surgical resection of pulmonary metastases has been associated with favorable survival outcomes in selected patients [113,120,129,130]. In one study, elevated AFP at the time of resection and the use of adjuvant chemotherapy were identified as independent risk factors for decreased survival after resection. However, these findings may reflect selection bias, as patients receiving adjuvant therapy often had larger or multifocal disease burdens, which could have independently influenced prognosis [113]. Despite extrahepatic recurrence accounting for a considerable number of cases, there is limited guidance regarding the management when resection is not feasible [131]. Systemic therapies, including targeted agents and immunotherapy, are increasingly being explored in this setting, although data remain scarce in the post-transplant population [114].

### 6.3. Systemic Therapies

Systemic therapies have significantly advanced the management of HCC, particularly in patients ineligible for surgical resection or with extrahepatic disease. However, most available data regarding these therapies are from the pre-transplant setting, and evidence supporting their use after LT is limited to isolated case reports and small series. As such, the oncologic risks and benefits of systemic therapy post-transplant remain an area of ongoing investigation [114].

Tyrosine kinase inhibitors (TKIs) initially began with sorafenib, which became the standard first-line treatment for advanced-stage HCC [132,133]. However, sorafenib was associated with considerable toxicity, leading to limited tolerability in some patients [134]. Recent treatment strategies have shifted, with lenvatinib now established as an alternative first-line therapy for patients with unresectable HCC [135]. In a phase III non-inferiority trial, lenvatinib demonstrated comparable median overall survival to sorafenib [135]. More recently, a single-center retrospective cohort study evaluated 120 patients who developed HCC recurrence after LT, of whom 56 TKIs, 42 were treated with sorafenib, and 14 with lenvatinib. The results showed that lenvatinib was associated with superior outcomes compared to sorafenib in this patient population [109]. However, lenvatinib is not without adverse effects. In a case series and a multicenter retrospective study involving patients with recurrent HCC after LT who were treated with lenvatinib, hypertension and fatigue emerged as the most commonly reported side effects [110,111].

In a non-transplant setting, immune checkpoint inhibitors (ICIs) have emerged as a promising class of systemic therapy in HCC, targeting immune regulatory pathways such as Programmed Cell Death Protein 1 (PD-1) (e.g., nivolumab), Programmed Death-Ligand 1 (PD-L1) (e.g., atezolizumab, durvalumab), and Cytotoxic T-Lymphocyte-Associated Protein 4 (CTLA-4) (e.g., tremelimumab) [136]. These agents have demonstrated significant efficacy in advanced HCC, particularly in combination regimens such as atezolizumab with bevacizumab or durvalumab with tremelimumab, which are now considered part of the first-line treatment for unresectable disease [137,138]. The IMbrave050 clinical trial demonstrated the superiority of atezolizumab–bevacizumab over active surveillance in preventing recurrence after curative resection or ablation [139]. However, in the post-LT setting, evidence for ICI use is limited to case reports and small series and remains controversial because of the substantial risk of allograft rejection. In a pooled analysis of 64 solid-organ transplant recipients, rejection occurred in 41% overall, including 7 of 19 liver transplant recipients (39%), with the highest rates seen with PD-1 agents (nivolumab 54%, pembrolizumab 39%); graft salvage was achieved in only ~29% of rejection episodes [112]. In a prospective LT series for recurrent HCC (*n* = 5), one patient died from rapid progression after a single nivolumab dose; another developed biopsy-proven moderate–severe acute liver rejection after the first dose that resolved with high-dose corticosteroids [125]. The four surviving patients were subsequently started on bevacizumab, all achieved stable disease, and remained on therapy at a median of 16 months, with longer survival versus regorafenib [125]. These data underscore that while ICIs may achieve disease control in carefully selected patients with post-LT recurrent HCC, the risk of acute rejection and graft loss is high and warrants close monitoring.

Despite these risks, ICIs may still be considered in selected LT recipients with recurrent HCC that is unresectable or refractory to other treatments, especially when the alternative is limited to supportive care [114]. The risk of rejection appears to be influenced by the timing of ICI administration, and a pre-transplant washout period of more than ~50 days has been suggested to potentially reduce this risk [137,140]. Emerging evidence also suggests that patients with distant metastases may benefit from these therapies, which have demonstrated improved outcomes compared to sorafenib. Given the delicate balance between oncologic benefit and immunologic risk, the use of ICIs in transplant recipients requires decision-making in a multidisciplinary fashion on a case-by-case basis. In addition to careful patient selection, close monitoring throughout the treatment is necessary [141].

## 7. Future Directions

In conclusion, post-LT HCC recurrence remains a major challenge with significant morbidity and mortality implications. For this reason, improving the outcomes of these patients requires a multifaceted approach, including pre-transplant risk stratification and an individualized post-LT surveillance protocol [102,103]. With the advancement in Omics technologies, in the near future, HCC LT selection criteria will most likely include biomarkers of tumor biology in association with tumor volume (number and size) [18]. At the same time, artificial intelligence (AI) remains a promising tool for the prediction of recurrence. Recent evidence has demonstrated the potential of machine learning models in accurately predicting HCC survival [142]. In line with this, a recent systematic review highlighted that AI-driven predictive models can support personalized risk assessment and guide treatment strategies to reduce recurrence by addressing patient-specific modifiable factors [143].

In addition, it appears that the focus on outcomes in transplant oncology is shifting from post-LT recurrence to post-LT survival. There are reports of using LT in highly selected patients with intrahepatic cholangiocarcinoma and colorectal cancer with liver involvement [144,145]. However, this approach may be limited by the balance between supply and demand. Therefore, other types of organ transplant, such as Donation after Circulatory Death (DCD), LDLT, and xenotransplant, may soon be considered more often in these settings [146].

There is a need to reach a consensus on the optimal timing and frequency of post-transplant imaging. In addition, surveillance strategies should be expanded to include broader imaging modalities to detect occult and extrahepatic recurrence. There is a pressing need for the development of new biomarkers to better predict recurrence [147]. Circulating DNA and RNA-based studies may also become important in the selection and monitoring of these patients [98,100,101]. HCC recurrence following liver transplant is associated with poor outcomes and very limited effective systemic therapies, such as chemotherapy or immunotherapy [141]. It appears that the current immunotherapy drugs are associated with high rates of post-LT rejections, and decisions regarding their use should be made on a case-by-case basis, carefully balancing oncologic benefits with immunologic risk [141]. As systemic therapies continue to evolve and become more targeted, they may offer better treatment options for extrahepatic metastatic HCC with less risk of rejection in the near future [114]. Further investigation is needed to address these knowledge gaps and guide evidence-based strategies for post-transplant care in HCC.

## Figures and Tables

**Table 1:** Location of Recurrence After Liver Transplantation for HCC and Treatment Strategies.

Location	Resectability	Treatment Options	Curative Intent
Intrahepatic (liver graft)	Resectable	Liver resection [5,104,105]	Curative
Intrahepatic (liver graft)	Unresectable	Thermal ablation (TA): (RFA/MWA) [106]	Curative in selected cases
		TACE [107]	Mostly non-curative
		Yttrium-90 (Y-90) radioembolization [108]	Mostly non-curative
		Systemic therapy (TKIs: sorafenib, lenvatinib, regorafenib, cabozantinib) [109,110,111]	Non-curative
		Immunotherapy (ICIs: nivolumab, pembrolizumab; durvalumab ± tremelimumab; atezolizumab + bevacizumab) [112]	Non-curative
Extrahepatic (lungs, bone, adrenal, LN, etc.)	Resectable/oligometastatic	Metastasectomy (e.g., lung resection) [113]	Curative in selected cases
Extrahepatic	Unresectable/multifocal	Systemic therapy (TKIs) [114]	Non-curative
		Immunotherapy (ICIs) [114]	Non-curative

TA: thermal ablation; RFA: radiofrequency thermal ablation; MWA: microwave ablation; TACE: transarterial chemoembolization; Y-90: Yttrium-90; TKI: tyrosine kinase inhibitors; ICIs: immune checkpoint inhibitors.

**Table 2:** Outcomes of Post-Transplant HCC Recurrence by Treatment Strategy.

Treatment	Study (Year)	*n* (Patients)	Recurrence Site	Median OS (mo)/Survival %
Surgical resection	Fernández-Sevilla 2017 [115]	22	Extrahepatic/Intrahepatic	35 mo (resected); 15 mo (non-resected)
	Kornberg 2010 [116]	7	Extrahepatic/Intrahepatic	65 mo (resected); 5 (non-resected)
	Faber 2011 [117]	27	Intrahepatic	1-, 3-, 5-y OS:96%, 70% and 42%
	Yoh 2021 [118]	430	Extrahepatic/Intrahepatic	1-, 3-, 5-y OS: 91.0%, 68.9%, and 55.1%
	Huang 2016 [106]	15	Extrahepatic/Intrahepatic	1-, 3-, 5-y OS: 92%, 51%, 35%
	Valdivieso 2010 [105]	8	Extrahepatic/Intrahepatic	33.2 mo after recurrence; RFS 21.2 mo
Pulmonary metastasectomy	Jeong 2021 [113]	52	Extrahepatic (lung)	75% 1-y, 43.5% 3-y, 33.9% 5-y
	Kim 2024 [119]	63	Extrahepatic (lung)	1-, 2-, and 5-y: 79.1%, 63.9%, and 35.6%
	Yoon 2010 [120]	45	Extrahepatic (lung)	40.7 mo; 5-y OS: 37%
Thermal ablation (RFA/MWA)	Bai 2021 [121]	297	Extrahepatic/Intrahepatic	1-, 3-, 5-y OS:91.9%, 71.2%, 58.7%
	Adwan 2023 [122]	40	Intrahepatic	1-, 3-, 6-y OS:97%, 80.3%, 60%
	Huang 2016 [106]	11	Extrahepatic/Intrahepatic	1-, 3-, 5-y OS: 87%, 51%, 28%
TACE	Wong Choi [123]	114	Extrahepatic/Intrahepatic	1-, 3-, 5-y OS: 77.8%, 53.6%, and 31.6%
	Hsieh 2014 [124]	11	Intrahepatic	6.6 mo (range 0.3–12.7); 1-y survival: 12.5%; all died ≤3-y
Regorafenib	Di Marco 2025 [125]	12	Extrahepatic/Intrahepatic	16.0 mo after initiation of 2nd-line therapy
Nivolumab/Bevacizumab	Di Marco 2025 [125]	4	Extrahepatic/Intrahepatic	5.8 mo after initiation of 2nd-line therapy
Everolimus + Sorafenib	Valdivieso 2010 [105]	5	Not detailed; included resected and unresectable	18.8 mo after recurrence
Lenvatinib	Magyar 2025 [109]	14	Extrahepatic/Intrahepatic	13.7–15.0 mo after TKI; 30.7 mo after recurrence
Sorafenib	Magyar 2025 [109]	42	Extrahepatic/Intrahepatic	7.8 mo after TKI; 18.1 mo after recurrence
Doxorubicin	Valdivieso 2010 [105]	2	Not specified in detail	16.3–21.7 mo

RFA: radiofrequency ablation, MWA: microwave ablation, TACE: transarterial chemoembolization; OS: overall survival.

## Abbreviations

The following abbreviations are used in this manuscript:

U7	Up-to-7 criteria
KC	Kyoto Criteria
AC	Asan Criteria
HALT	Hazard Associated with Liver Transplantation for HCC Criteria
MC	Milan Criteria
UCSF	University of California San Francisco
UNOS	United Network for Organ Sharing
AFP	Alpha-fetoprotein
LDLT	Living Donor Liver Transplants
DCD	Donation after Circulatory Death
HCC	Hepatocellular Carcinoma
ctDNA	Circulating tumor DNA
EVs	Extracellular vesicles
LT	Liver transplantatio8n
MASLD	Metabolic Dysfunction-Associated Steatotic Liver Disease
MVI	Microvascular Invasion
LRT	locoregional therapy
ILTS	International Liver Transplantation Society
AASLD	The American Association for the Study of Liver Diseases
ICI	Immune checkpoint inhibitors
CNIs	Calcineurin Inhibitors
MMF	Mycophenolate mofetil
mTOR	Mammalian target of rapamycin
PD-1	Programmed cell death protein 1
PD-L1	Programmed Death-Ligand 1
CTLA-4	Cytotoxic T-Lymphocyte-Associated Protein 4
Y90	Yttrium-90
TARE	Transarterial Radioembolization
TACE	Transarterial Chemoembolization
TKI	Tyrosine kinase inhibitors
